# Physicochemical Analysis of Sediments Formed on the Surface of Hydrophilic Intraocular Lens after Descemet’s Stripping Endothelial Keratoplasty

**DOI:** 10.3390/ma13184145

**Published:** 2020-09-17

**Authors:** Dorota Tarnawska, Katarzyna Balin, Maria Jastrzębska, Agnieszka Talik, Roman Wrzalik

**Affiliations:** 1 Institute of Biomedical Engineering, Faculty of Science and Technology, University of Silesia, 41-200 Sosnowiec, Poland; dorota.tarnawska@us.edu.pl; 2 Department of Ophthalmology, District Railway Hospital, Panewnicka 65, 40-760 Katowice, Poland; 3 August Chełkowski Institute of Physics, Faculty of Science and Technology, University of Silesia, 41-500 Chorzów, Poland; maria.jastrzebska@us.edu.pl (M.J.); agnieszka.talik@smcebi.edu.pl (A.T.); roman.wrzalik@us.edu.pl (R.W.)

**Keywords:** hydrophilic intraocular lens, opacification, sedimentation, DSAEK, AFM microscopy, XPS and TOF–SIMS spectroscopy

## Abstract

An intraocular lens (IOL) is a synthetic, artificial lens placed inside the eye that replaces a natural lens that is surgically removed, usually as part of cataract surgery. The opacification of the artificial lens can be related to the formation of the sediments on its surface and could seriously impair vision. The physicochemical analysis was performed on an explanted hydrophilic IOL and compared to the unused one, considered as a reference IOL. The studies were carried out using surface sensitive techniques, which can contribute to a better understanding of the sedimentation process on hydrophilic IOLs’ surfaces. The microscopic studies allowed us to determine the morphology of sediments observed on explanted IOL. The photoelectron spectroscopy measurements revealed the presence of organic and inorganic compounds at the lens surface. Mass spectroscopy measurements confirmed the chemical composition of deposits and allowed for chemical imaging of the IOL surface. Applied techniques allowed to obtain a new set of information approximating the origin of the sediments’ formation on the surface of the hydrophilic IOLs after Descemet’s stripping endothelial keratoplasty.

## 1. Introduction

Cataract surgery is the one of the most common surgeries in the world and is generally considered as a safe procedure. Patients with concomitant senile cataract and dysfunction of corneal endothelium, the most posterior part of the cornea, are at increased risk of irreversible corneal edema and loss of its transparency after cataract surgery. In the event of such a complication, the only effective treatment is corneal transplant (keratoplasty). Regardless of whether the cataract surgery is performed sequentially or simultaneously with corneal transplantation, at present, one of the posterior keratoplasty techniques is used in these patients. A few years after the introduction of posterior lamellar keratoplasty techniques, disturbing reports began to appear about the formation of deposits on commonly used intraocular lenses, which significantly deteriorate vision. The occurrence of this phenomenon was associated with intraocular gas injection used in these techniques. Despite the many cases reported, most of them are based only on clinical descriptions, while a few studies containing chemical analysis of sediments do not provide a chemical or physical explanation for this phenomenon.

Here, we present the results of physicochemical analysis of the anterior surface of two hydrophilic intraocular lenses: one that was explanted from the patient’s eye and the other brand new, unused lens. The study was carried out to find out the reasons for the sediment formation on hydrophilic intraocular lenses in patients after Descemet’s stripping endothelial keratoplasty (DSAEK). By identifying chemical bonds between the elements and linking detected compounds with the morphology of the unused and explanted hydrophilic lens surfaces, we have expanded the scope of information available in this subject, and brought a methodology that can contribute to a better understanding of the main causes of the sediment formation.

### 1.1. Medical Background

During modern cataract surgery, an intraocular lens (IOL) with appropriate optical power is implanted to replace the removed opaque natural lens. Currently available IOLs are made of various materials, including silicon, hydrophobic acrylic, and hydrophilic acrylic. Hydrophobic acrylic IOLs are the most commonly used thanks to the high refractive index and relatively low posterior capsule opacification rate [1]. In turn, hydrophilic acrylic IOLs cause less postoperative inflammation because of better biocompatibility with ocular tissues [2]. Opacification of the IOL caused by the formation of sediments on its surface is an uncommon postoperative complication, but it is considered a serious problem. This can lead to significant visual disturbances and thus the need for IOL explanation [3,4,5].

The opacification has a very characteristic appearance as it is localized on the anterior surface of the IOL and limited to the pupil zone. The cause of this complication often remains unclear. It is known, however, that it occurs more often after surgery involving the use of gas or air inside of the eye [5,6,7]. Because DSAEK as well as other techniques of posterior lamellar corneal transplantation have become common surgeries, they are currently the leading procedures associated with late-onset IOL opacification [3,4,8]. The incidence of hydrophilic acrylic IOL opacification after DSAEK ranged from approximately 5% [9] to 9% [10] in larger series of patients, while opacification of the hydrophobic IOLs after this procedure has been reported only sporadically [10].

DSAEK is a partial thickness cornea transplant procedure that replaces only the posterior layer of the cornea. The main advantages of DSAEK when compared with conventional, full-thickness penetrating corneal transplantation are faster visual rehabilitation, better visual outcomes with minimal change in corneal surface topography or refraction, and lower risk of graft rejection [11,12]. This technique includes the selective removal of the patient’s Descemet’s membrane and endothelium followed by transplantation of the donor corneal lenticule, which consists of a thin layer of posterior stroma, Descemet’s membrane, and the endothelium. The donor graft, which is about 100–200 microns thick, is attached to the posterior surface of the recipient’s cornea, only using an air or gas bubble, injected into the anterior chamber of the eye. A patient undergoing DSAEK is already pseudophakic (has an IOL implanted in the eye) or cataract surgery is performed simultaneously. Intraocular lens opacification after DSAEK is a serious complication that causes a significant decrease of visual acuity and may require IOL exchange [3]. Exchange of the IOL in patients after DSAEK is particularly dangerous, because it has a strong negative impact on long-term graft survival and may also cause other ocular complications [13]. Therefore, in patients with less advanced opacity, management is often limited to observation only [4]. The key factors in IOL opacification in patients after DSAEK that statistically increase the risk of this complication are repeated exposure to intracameral air or gas [3,4,10], raised IOP [10], along with the presence of hydrophilic acrylic lens. Besides air contact, prolonged breakdown of the blood–aqueous barrier could also play a role in the induction of IOL opacification [4,5,6]. Therefore, in patients with endothelial dystrophy, hydrophilic IOL implants are not recommended during cataract surgery, as it is likely that DSAEK will be necessary in the future [10,14].

While hydrophilic acrylic IOLs are not very popular in the United States, they are still widely used in many European countries, including Poland and Germany [15]. Therefore, the complication may still potentially arise in a certain group of pseudophakic patients who have already received hydrophilic acrylic IOL models and who will need DSAEK or Descemet’s membrane endothelial keratoplasty (DMEK) in the future owing to acquired endothelial dysfunction. Thus, despite the fact that, because the problem of IOL opacification has been known, patients with existing or impending endothelial disorders have been implanted with hydrophobic lenses, the issue still exists.

**Figure 1:**
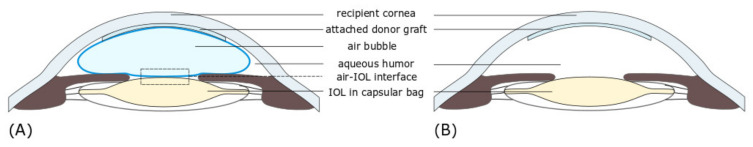
Schematic diagram of cross section through the anterior chamber of the eyeball after Descemet’s stripping endothelial keratoplasty (DSAEK), showing the location of corneal lamellar graft attached to posterior surface of the host cornea, an air bubble, and the intraocular lens (IOL) when the patient is laying face up. (**A**). The injected air fills 90 percent of the volume of the anterior chamber, supporting the graft. The area of contact between air and IOL (frame) corresponds to the area shown in Figure 1B. (**B**) After the air bubble has been absorbed, the anterior chamber is completely filled with aqueous humor.

Figure 1A shows the diagram of implanted IOL and air bubble supporting the transplant.

IOL opacification can not only decrease the visual acuity, but also worsen the quality of vision. For less opacified IOLs, the only noticeable symptoms may be the reduced perception of brightness and contrast. “Blurred” vision is usually associated with moderate opacity, while dense opacification can ultimately result in a significant decrease of visual acuity. Some recent studies described the quantitative analysis of the optical quality of the explanted IOLs with the use of an optical bench. The measurements showed a deterioration of the optical quality with a marked decrease of the modulation transfer function (MTF) values across all spatial frequencies and degradation of United States Air Force 1951 resolution target images [3,16,17]. Other experiments showed an increased light scattering and a decreased light transmittance through explanted opacified IOLs, which explains the subjective symptoms in patients such as glare and halos, reduced contrast sensitivity, and decreased visual acuity [18,19,20]. Łabuz et al. show that there is a proportional relationship between straylight and the morphological parameters of the IOL opacification, that is, the area and density of sediment. This observation may explain the existing differences in the decreased optical quality between the affected patients that is independent of their visual acuity [21].

### 1.2. Physicochemical Analysis of Reported Cases

Various physicochemical studies have been applied to the analysis of hydrophilic IOLs’ opacification. Among them, light and electron microscopy, as well as elemental or molecular surface analytic techniques, demonstrated that the opacification, similar to presented in this work, was related to calcium phosphate precipitation or adsorption of fatty acids and proteins on the IOLs’ surface [22,23,24,25]. Sediments observed on IOLs are, in most described cases, distributed on the anterior and posterior surface of the optical zone, limited to the pupillary zone, but rarely appear in the peripheral part and haptics [26]. Light and electron microscopy studies indicate that observed sediment arises from the enlargement of smaller and deeper subsurface lesions [3]. Electron microscopy studies for some cases indicate that observed sediments may penetrate the IOLs near the surface region [3,5,27]. The circular shape opacification on IOLs can be also associated with an air injection into the anterior chamber of the eye following DSAEK or Descemet’s membrane endothelial keratoplasty (DMEK), another technique of posterior lamellar keratoplasty, within the margins of the anterior capsulorhexis (round central opening in the anterior lens capsule), and is related to the area where the air bubble touches the IOL [9,10,28].

The elemental composition of the IOLs’ sediments was based on the spectroscopic analysis using X-ray energy-dispersive (EDS) and X-ray photoelectron (XPS) spectroscopies. For instance, EDS confirmed the presence of calcium phosphate, thus proving that opacification was attributed to calcification [29,30,31], whereas detected sodium and chlorine were recognized as artefact from the saline solution [32]. However, the XPS analysis performed by Yang et al. [24] revealed that the main components of IOL sediments were calcium and silicone. Their observation confirmed that silicon has a role in the calcification of the hydrophilic intraocular lens, as postulated earlier by Dorey et al. [33], and could induce calcium-phosphorus deposits on IOL [34]. Most published case reports rarely focus in detail on physicochemical analysis, including the chemical composition and processes leading to the formation of sediment on the surface of the IOLs. In general, as described in many reports, the opacification most likely originates from a combination of different factors related to IOLs’ manufacturing, packaging, surgical techniques, and lastly patient conditions. The opacification related to the calcification has been classified into two categories, primary and secondary [35], although other types of categorization of tissue artifacts associated with pseudo calcifications are also used. The primary opacification is caused by IOL defects that appear to originate during manufacturing and packaging processes. In such cases, the observed opacification occurs as a result of sediment formation, which in the next step penetrates the surface of the lens. Meanwhile, the secondary calcification refers to sediment deposits observed on the surface of the IOLs and is related to the environmental conditions to which IOLs have been exposed, including, for example, the breakdown of the aqueous–blood barrier occurring as a result of pre-existing patient conditions. Recent studies emphasize the importance of blood–aqueous barrier breakdown [36,37,38], the coexistence of pro-inflammatory condition and repeated gas injection [39], together with the change in the hydration of the hydrophilic lens [38] in the sediment formation process. However, the authors of many papers repeatedly emphasize the lack of a well-defined mechanism of sediment formation [40,41].

Because many variables may affect the sedimentation process, a common mechanism for different types of IOLs still has to be examined. Here, we present a most probable mechanism of sediment formation based on one specific explanted hydrophilic IOL. Unrevealed, through physicochemical surface analysis, the probable cause of sediment formation may allow the development of materials and methods to prevent sedimentation of the hydrophilic IOLs.

## 2. Experimental Details

### 2.1. Case Report

A 56-year-old man was diagnosed with bilateral Fuchs’ endothelial dystrophy, endothelial decompensation after cataract surgery with IOL implantation in his right eye, and an initial cataract in his left eye. An uneventful DSAEK was performed on his right eye. After donor graft insertion, an air bubble was injected into the anterior chamber for graft apposition and kept for 5 min at the pressure between 20 and 30 mmHg, followed by exchanging air with a balanced salt solution. At the end of the procedure, a residual air bubble filling 50% of the anterior chamber volume was left. On the second postoperative day, the graft was partially detached, so it was decided to re-bubble the anterior chamber with air. After re-bubbling, the graft remained well centered and completely attached. Postoperative standard medications included fluoroquinolones eye drops four times a day for 2 weeks and prednisolone acetate eye drops 1% four times daily for a month, then to be tapered accordingly. After the initial visual improvement, at five months post-operatively, the patient began complaining about worsening of visual acuity. At that time, the cornea was slightly edematous with no sign of postoperative anterior chamber inflammation. However, ten months following DSAEK, the corneal graft became cloudy and corneal decompensation was diagnosed. Upon slit-lamp examination, in addition to corneal decompensation, central IOL opacification confined to the pupillary zone of the anterior IOL surface was found. The patient subsequently underwent penetrating keratoplasty (PKP) with IOL exchange. During the surgery, the opacified IOL was explanted from the bag and a new IOL was implanted in the ciliary sulcus because of suspected damage to the capsular zonules.

The explanted IOL was then imaged by optical microscopy, atomic force microscopy, photoelectron spectroscopy, and mass spectroscopy to investigate the morphology of the surface opacification and its chemical composition. The patient was operated on and followed up at the Department of Ophthalmology, District Railway Hospital in Katowice. Both surgeries were performed by the same surgeon (D.T.).

### 2.2. Sample Preparation

The analysis was performed on two IOL implants. As a reference sample, the unused same type lens (C-flex 570C, Rayner Intraocular Lenses Ltd., East Essex, UK), stored in the original packaging, was used. The C-flex lenses are single piece posterior chamber IOLs that are designed to be surgically implanted into the capsular bag of the human eye for the visual correction of aphakia in patients in whom a cataractous lens has been removed with phacoemulsification. They are made from a hydrophilic acrylic co-polymer (Rayacryl), which was designed to reduce the problems of silicone oil adhesion, and thus silicon oil induced opacification [42]. Before the spectroscopic measurements, the reference lens was taken out of the packaging, dried, and later placed in ultrahigh vacuum (UHV) conditions. The second C-Flex IOL, with well visible surface sediment, after explanting from a patient’s eye, was kept dry in a tightly closed box placed in a refrigerator for several days before placing it in the UHV chamber.

### 2.3. Applied Techniques

Two different techniques, XPS and time of flight secondary ion mass spectrometry (TOF–SIMS), have been selected for the studies of the chemical composition of IOLs. Application of these techniques allows the detection of organic and non-organic compounds on the surface of the material being examined, and here, indirectly allows studying processes that occurred at the interface of the implant surface and surrounding tissue and fluids. Microscopic measurements were performed only for the explanted IOL.

#### 2.3.1. Atomic Force Microscopy (AFM)

AFM is a high-resolution scanning probe microscopy that uses a very sharp tip to scan the surface of a sample. Measuring the tip–sample interaction enables quantitative and qualitative measurement of various properties such as morphology, elastic properties, local conductivity, and other material properties.

All AFM imaging was performed under ambient conditions using a NanoWizard III AFM (JPK Instruments, Berlin, Germany) mounted on Axio Observer A1 inverted microscope (Zeiss, Oberkochen, Germany) equipped with 10×, 20×, and 40× objectives and accessories for differential interference contrast (DIC) observation. To reduce the noise floor and acoustic vibrations, the system was placed on a halcyonics_i4 active vibration isolation table (Halcyonics, Goettingen, Germany). The AFM head was equipped with a 15 μm z-range linearized piezoelectric ceramic scanner and an infrared laser. The setup was used in closed height feedback mode (specified positioning accuracy of better than 0.1 nm). Calibration of the scanner in the XY plane was verified by scanning 3 μm grating (TGG1, NT-MDT Spectrum Instruments, Moscow, Russia) in tapping mode at a 1 Hz scan rate. Measurements of the grating pitch were within 1.5% of the specified value. Measurements of grating depth (TGZ3, NT-MDT) from the capacitive sensor channel on the Z scanner were within 3% of the specification. Tapping mode AFM images were acquired using Tap300-G BugetSensors probes (nominal force constant 30 N/m, resonance frequency 300 kHz). Images were collected with an amplitude set point typically around 0.4 V, within a few tens of millivolts of the free amplitude, corresponding to an amplitude of approximately 15 nm and a mean tip–sample force on the order of some hundreds of pN. Height and error signal were recorded in both trace and retrace directions with a resolution of 512 points per line at a scan rate from 1 Hz to 0.5 Hz (depending on the size of measurement area). Image analysis was performed with ImageJ software (U.S. National Institutes of Health, Bethesda, MD, USA) or the open source software Gwyddion (version 2.55), Brno, Czech Republic.

#### 2.3.2. The X-ray Photoelectron Spectroscopy (XPS)

In the XPS spectroscopy, irradiating a sample with monochromatic X-rays causes the emission of photoelectrons whose energies are characteristic of the elements within the sampling volume. The intensity and position of characteristic XPS lines determine the concentration and local chemistry or bonding environments of the detected atom. XPS can identify the presence of a particular element unambiguously, as well as identify its bonding environment.

XPS measurements were executed with the use of a VG Scienta/Prevac photoelectron spectrometer (VG Scienta AB, Uppsala, Sweden and PREVAC sp. z o.o., Rogow, Poland). Monochromatic Al Kα radiation of the energy of 1486 eV was used for excitation of photoelectrons from the sample surface. The photoemission spectra were collected in a wide binding energy range (−2 to 1400 eV) and in the ranges representative for the characteristic photoemission lines of individual elements detected on IOLs’ surface. Analysis was carried out using PHI MultiPak (v.9.6.0.15) software (ULVAC PHI, Chigasaki, Japan). XPS has allowed obtaining information about the chemical composition of the sample with the accuracy of detection of 0.1 atomic percent from the surface with a thickness of ~3 nm.

#### 2.3.3. Time of Flight Secondary Ion Mass Spectrometry (TOF–SIMS)

Further analysis was realized with the use of TOF–SIMS, a surface analytical technique that rasters a high-energy ionizing beam over a predetermined area, producing secondary ions from a sample surface, in a sputtering process. Analysis of these secondary ions provides information about the elemental and molecular species present on the surface of the characterized material.

TOF–SIMS measurements were carried out with the use of TOF–SIMS 5 (ION-TOF GmbH, Munster, Germany) reflection-type spectrometer, equipped with bismuth liquid metal ion gun, Bi_3_^+^ of energy of 30 keV, and current of about 0.5 pA. Positive secondary ions spectra and distribution maps for selected ions were collected by rastering the ion beam across predetermined areas. The surfaces of the samples were cleaned with the use of DC current bismuth ion beam and cesium beam (2 kV, 100 nA, Cs dose 1.2 × 10^18^ ions/cm^2^) in order to remove the surface contaminations. It has to be mentioned that using DC bismuth beam and Cs beam to the surface cleaning rapidly deteriorates organic signals; however, in the case of the experiment performed, even after long sputtering, it was still possible to detect signals from the organic components of the explanted IOL surface. The data were collected before and after the cleaning. The flood gun was used in order to compensate surface charging. Analysis was carried out using SurfaceLab6 software (IONTOF GmbH, Munster, Germany). TOF–SIMS allowed even more accurate chemical analysis (associated with a higher detection limit and lower sampling depth of about 0.4 nm, high detection limits for elements) combined with chemical imaging of the surface of the analyzed implants. XPS and TOF–SIMS studies were performed at room temperature, under ultra-high vacuum.

#### 2.3.4. Experimental Procedures for the Spectroscopic Measurements

The following approach was adopted for the IOLs spectroscopic studies. Firstly, knowing the history of both samples, we considered possible contamination of the IOLs. Because both IOLs remained exposed to air, their surface was covered with a thin layer of surface contamination, which, taking into account the sampling depth of used techniques, can affect the obtained results. Secondly, we consider the morphology of a visible sediment, mainly the thickness of the sediment observed on the surface of the explanted IOL, and its distribution on the analyzed areas. Thirdly, possible contamination of the explanted IOL with a biofilm, which may appear on the IOL surface after removing it from a human eye, was considered.

**Figure 2:**
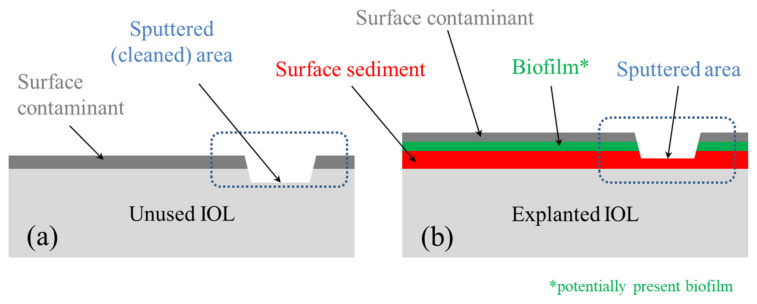
Schematic representation of cross section of characterized IOLs: (**a**) for the unused (reference) IOL and (**b**) for the explanted IOL. The diagrams are a section of the area marked in Figure 1.

Knowing these factors, the XPS technique, as the one with greater sampling depth, was applied on contaminated surfaces, to obtain information that includes both surface contamination and a small volume of the IOL near the surface. In contrast to XPS, in the case of more surface sensitive TOF–SIMS spectroscopy, the measurements were performed on contaminated and cleaned surfaces of both IOLs (in Figure 2, the cleaned area marked with a frame with dashed lines). For both techniques, the region where the sediment partially covered the explanted IOL was selected. For more details, see Comment S1, Table S1 and Figure S1a,b in Supplementary Materials.

Our study solely involves laboratory analyses of IOL explant. No additional procedures on humans or animals were performed. Informed consent and ethics committee approval were thus not required.

## 3. Results

### 3.1. Morphology

The first step of the analysis of the IOL was focused on imaging of the observed sediments; the general distribution of sediments on the IOL surface as well as the nano- and microstructure of observed sediments were determined using optical microscopy and AFM.

**Figure 3:**
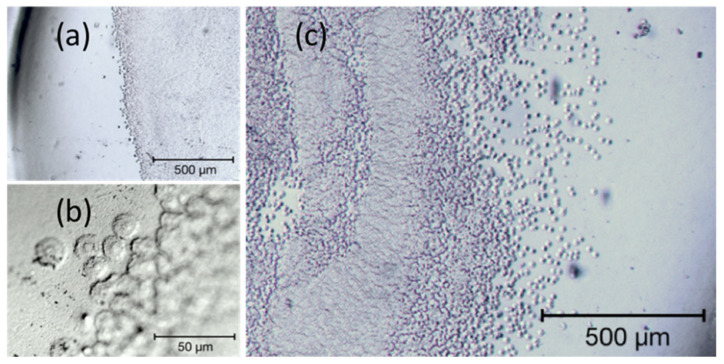
Selected areas of the explanted IOLs’ surface in bright-field microscopy. (**a**,**c**) shows two different partially sedimented regions of the lens surface, (**b**) magnified edge of the sedimentation with well-defined round shaped sediments.

As shown in Figure 3, the larger part of the outer surface of the IOL (below 50%, Figure 3a) is covered with embedded material, being the subject of the study. It is accumulated mainly in the central part (in the area of the pupil of the eye, see Figure 3a,b), which makes the lens almost opaque. On the edge of the sediment area, there are visible round structures with a diameter of several micrometers (Figure 3c), the number of which increases towards the center of the IOL. Their distribution is not even, and their number increases significantly towards the center, so that they merge into an almost homogeneous surface (see the central part of Figure 3b). The morphology of the sediment is different than in the case of snowflake structures observed for poly(methyl methacrylate PMMA intraocular lenses, in which the degeneration of the lens surface was related to the UV exposure of the IOL [43] and different than plate-like deposits observed for silicone IOLs [44]. The sediment distribution of the analyzed lens is similar to that observed on the hydrophilic intraocular lenses in patients who required additional re-bubbling (intracameral air injection) to reattach the donor graft [14].

**Figure 4:**
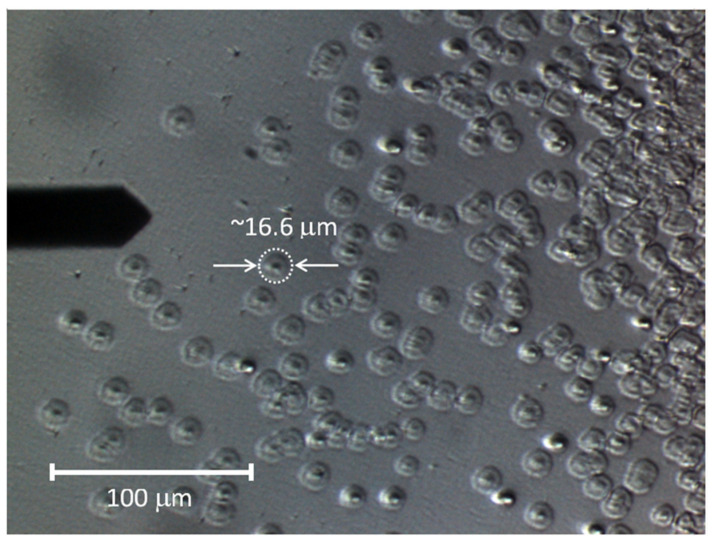
Differential interference contrast (DIC) image of the surface of explanted IOL.

The characteristic structures of the sediments are more visible in the DIC image, Figure 4, obtained using the optical microscope constituting the base of the AFM apparatus (note the AFM cantilever on the left-hand side of the micrograph). On the left side of the image, round, conical structures (hereinafter referred to as islands) are separated from each other. Their number increases in the middle part of the image, so that some of them touch each other, but their round shape is still clearly visible. Towards the lens center (in the right part of the image), they create an almost uniform, undulating surface (see the central part of Figure 3b).

**Figure 5:**
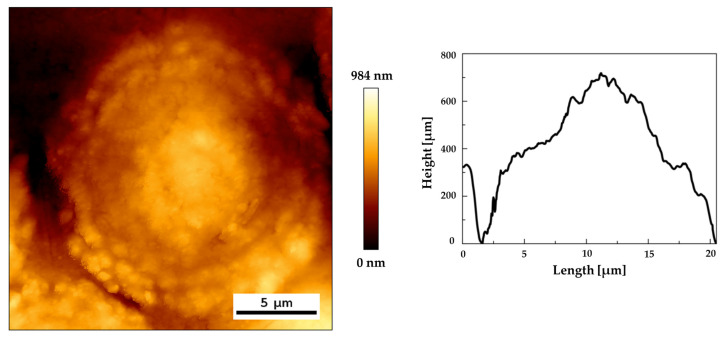
A tapping mode overview image of single sediment: (**a**) height image and (**b**) height profiles along lines.

The characteristic shape of a single sediment structure is shown in Figure 5, presenting an AFM height image and height line profiles. The conical, central part of the structure and its quickly falling edges are clearly visible. Statistical analysis of DIC and AFM images allowed to determine the average diameter and height of the islands as well as their standard deviations. The diameter values are 16.56 ± 2.55 µm for DIC and 15.32 ± 2.19 µm for AFM (for sample size n = 30). The height determined on the basis of the AFM measurements is 550.2 ± 68.5 nm.

### 3.2. Chemical Composition

#### 3.2.1. Surface Analysis by XPS

On the basis of the carried identification of the XPS survey spectra, the following elements were detected on the surface of both IOLs: carbon, nitrogen, oxygen, sodium, and silicon. However, it is noticeable that, in the explanted IOL, low quantities of calcium, chlorine, and sulfur were also found, while for the reference IOL, a trace of fluorine was detected. The detailed chemical composition and atomic concentrations are combined for both lenses in Table S2 in Comment S2 of Supplementary Materials.

**Figure 6:**
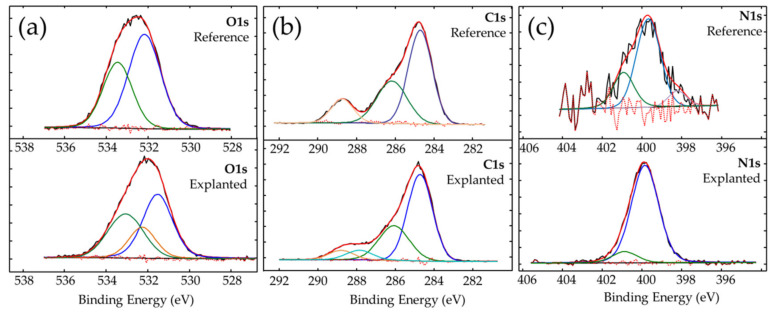
High resolution photoemission spectra of (**a**) oxygen O1s, (**b**) carbon C1s, and (**c**) nitrogen N1s for reference and explanted IOLs.

In order to determine the source of the origin of the detected elements, their chemical states were analyzed by detailed analysis of high-resolution photoemission lines. In this section, we show only the XPS spectra of the most prominent elements—oxygen, carbon, and nitrogen (see Figure 6); however, a similar analysis was performed for the residual detected elements. Analysis of the calcium photoemission line in the explanted IOL indicates the presence of calcium oxide, CaO (which is unlikely because of stability in the aquatic environment) [45], or more probably calcium carbonate CaCO_3_ [46]. The analysis of the sodium line, a relative strong line at 1071 eV, together with O1s line confirmed the presence of the Cl-O bond in Na environment, like in the NaClO_3_ compound [47]. The location of the chlorine line, detected in explanted IOL, indicates the presence of amine compounds, which is also evident in the oxygen or carbon lines described below. In the case of sulfur, the chemical state at binding energy of 163.52 eV corresponds to the phenylene polysulfide, [-C_6_H_4_-S-], material widely used in the production process of thermoplastics [48]. The second chemical state of sulfur at a binding energy of 168.13 eV most probably corresponds to sulfur dioxide SO_2_ [49].

The analysis of the oxygen O1s photoemission line shows, for both lenses, two different types of bonds between oxygen and carbon: C-O and C=O on the surface of both IOLs. The chemical state of oxygen in which C-O bonds were found is associated to the presence of surface contaminants. The chemical state at binding energy of 533.54 eV for the reference IOL indicates a double bond between oxygen and carbon and is related to the presence of poly (methyl methacrylate) (533.6 eV) [50], and can be associated with the material from which the IOL was made. The chemical state of oxygen at a binding energy of 533.09 eV, detected for explanted IOL, indicates the presence of oxygen and carbon double bonds and, according to the literature [51], can be associated with acrylic glass. In the case of the explanted IOL, an additional line at 532.41 eV was detected. It is most likely derived from sodium chlorate (NaClO_3_), for which the binding energy value is 532.40 eV [47]. The increased amount of nitrogen and carbon in explanted IOL (see Figure 6 and Table S2, Supplementary materials) is probably due to the presence of organic compounds on the lens surface, which should be expected after a long residence time of the IOL in the human eye.

The analysis of the carbon C1s line confirms the presence of carbon in specific chemical states; the presence of C-O, C=O and characteristic for carboxylate O-C=O bonds was observed on the surface of both examined IOLs. In addition, the chemical state of carbon with a binding energy of 287.48 eV was detected on explanted IOL. On the basis of literature reports, it can be linked to organic compound-polyaniline (-C_6_H_5_NH-)n [52]; one of the detected in explanted nitrogen states (peak at 399.82 eV) is, according to that paper [52], also assigned to that compound. However, as polyaniline is not expected to be present in such environment, we rather consider information from that reference as an indication of the presence of the bond between the carbon and NH group (-C-NH-C-), like in secondary amines or pyrrole ring. Such a pyrrole ring has, for example, tryptophan, detected using TOF–SIMS spectrometry. The same (-C-NH-C-) group can be observed in melanin, which has been detected on the surface of other IOLs as a result of mechanical malfunctions of the iris [53,54]. Here, the presence of melanin is most likely the result of inferior peripheral iridectomy (surgical removal of a very small part of the iris), which is usually routinely performed during DSAEK. Peripheral iridectomy allows the flow of aqueous humor when the pupil path is blocked by an air bubble in the period immediately after DSAEK. During this procedure, a certain amount of melanin-containing pigment cells are released from the iris pigment epithelium. Analysis of TOF–SIMS mass spectra additionally indicates the presence of detected amino acids, methionine and valine (see Table S3 and Figure S2 in Comment S3 of Supplementary Materials), among other amino acids present in aqueous humour [55,56]. Trace amounts of second detected chemical state of nitrogen (see Figure 6c low intensity N1s line for explanted IOL) are related to either the imide derived from amine group [57] or the organic acids [58].

Some differences were also observed in the amount and shape of silicon Si2p photoemission lines. An increased amount of silicone detected at 101.59 eV is associated with silicone nitrite [59], and a slightly increased amount of nonstoichiometric silicone oxide at 103.21 eV [60] was observed on the surface of explanted IOLs. For MemoryLens, SC60B-OUV and Aqua-Sense hydrophilic lenses, the silicon contaminations are considered as an important factor in the opacification process [34]. It has also been found that different types of viscoelastic materials exhibit different calcification properties. Under experimental conditions, it was shown that, for Viscoat or Amvisc Plus, but not OcuCoat, the nucleation and growth of octacalcium phosphate crystallites on hydrophilic IOLs was induced as a result of the adsorption of hydrophobic cyclic silicone [30]. However, in the case of Rayacryl C-Flex lenses, studied by us, the base material was designed to reduce the potential influence of the silicon on sedimentation process. Apart from a slight increase of the silicon amount on the explanted lens surface, we have not found convincing evidence that silicone is the only cause of the opacification, because more significant changes in the chemical composition were observed. Considering the cases described in some papers [30,34] and studies of the H60M, SC60B-OUV, MemoryLens, and Aqua-Sense IOLs in which silicon was not detected in sediments [29], the silicon influence on the sedimentation process most likely depends on the material from which the lens was made. However, it has to be mentioned that the presence of the silicone in the case of studied IOL may also result from the surface contamination of the surface of the explanted lens, which can appear after lens explantation.

#### 3.2.2. Morphology and Surface Analysis by TOF–SIMS

Further analysis of the elemental and molecular composition of contaminated and cleaned surfaces of the both IOLs was based on the TOF–SIMS spectra and derived from the distribution maps of individual ions. The combination of analysis of contaminated and cleaned surfaces allowed to link the chemical composition of the IOLs with their surface morphology. Mass spectra analysis revealed the presence of the following elements on the IOLs’ surface: hydrogen, lithium, carbon, oxygen, magnesium, sodium, aluminum, silicon, potassium, calcium, sulfur, and copper. Additionally, traces of iron and phosphorus were found on the surface of the explanted IOL.

**Figure 7:**
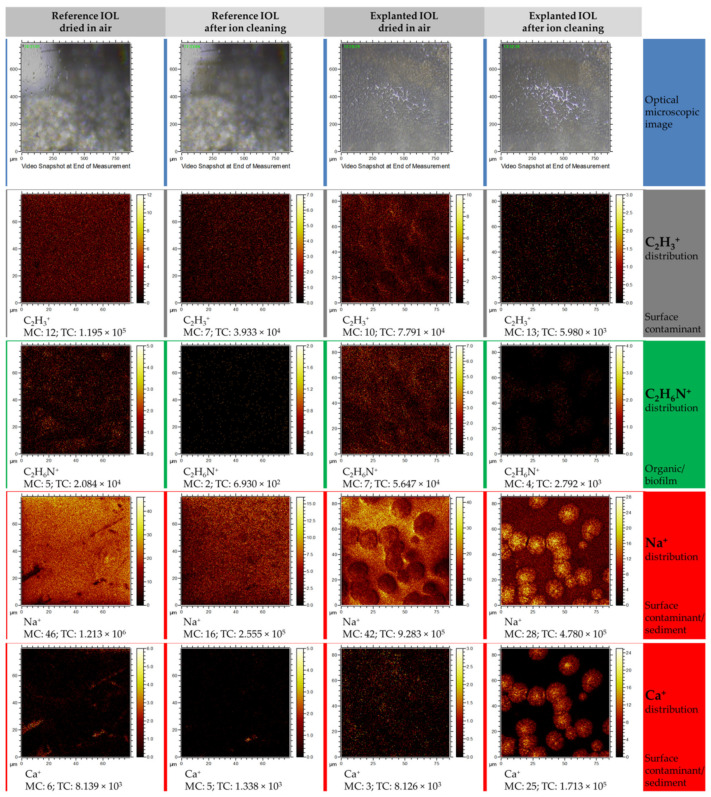
Time of flight secondary ion mass spectrometry (TOF–SIMS) distribution maps of selected elemental and molecular ions obtained in positive polarity mode measurements. Each column represents a set of distribution maps of C_2_H_3_^+^, C_2_H_6_N^+^, Na^+^, and Ca^+^. The first and second columns represent the ions’ distribution for the reference lens before and after cleaning procedure, respectively. The third and fourth columns show before and after cleaning of the explanted lens, respectively.

Below, the microscopic image of analyzed areas, 800 × 800 μm in size, was combined with reconstructed distribution maps from the 80 × 80 μm area located in the center of the microscopic image (see Figure 7). Preliminary analysis of microscopic images allows us to assess that the turbidity visible in the explanted IOL is the result of the appearance of opaque sediment on the IOL. Therefore, an area with well visible sediment, only partially covering the IOL surface, was selected for the analysis.

To present the results of the elemental and chemical mapping, only the most significant distribution maps were selected. Analysis of the C_2_H_3_^+^ ion distribution before and after surface cleaning indicates that this ion, as expected, is a component of the surface contamination layer, which is partially (the reference IOL) or almost completely removed (explanted IOL) after the cleaning process. Similarly, in the case of the organic ion C_2_H_6_N^+^, whose distribution differs from the distribution of the C_2_H_3_^+^ ion, but is the same as in the case of C_2_H_3_^+^, it creates a surface layer that can be easily removed after surface cleaning. The presence of C_2_H_6_N^+^ is most likely related to the presence of the biofilm on the sediment surface. The occurrence of Na^+^ ion may be associated with both the contamination of the entire lens surface and the sediment itself. Sodium accumulates in areas with a diameter of ~15 μm, which partially cover the analyzed area and were also observed in AFM measurements. In the case of a reference lens, its source is mainly sodium chloride, as presented in Guan et al. study [30], which is part of the liquid in which it is stored (physiological saline). However, for explanted IOL, it comes from aqueous humour, which contains a significant concentration of various minerals and electrolytes. Regarding the distribution of Ca^+^ ions, it can be seen on the maps registered for the reference lens that calcium ions accumulate in irregular areas, most likely related to the mechanical microdefects of IOLs’ surface. After cleaning of the surface, the amount of calcium in these areas decreases or calcium is completely removed. However, in the case of explanted IOL, trace amounts of calcium can be observed in the contaminated surface layer, and after removing the thin contaminant layer, it is clear that the surface containing the circular sodium-related structures also contains large amounts of calcium.

**Figure 8:**
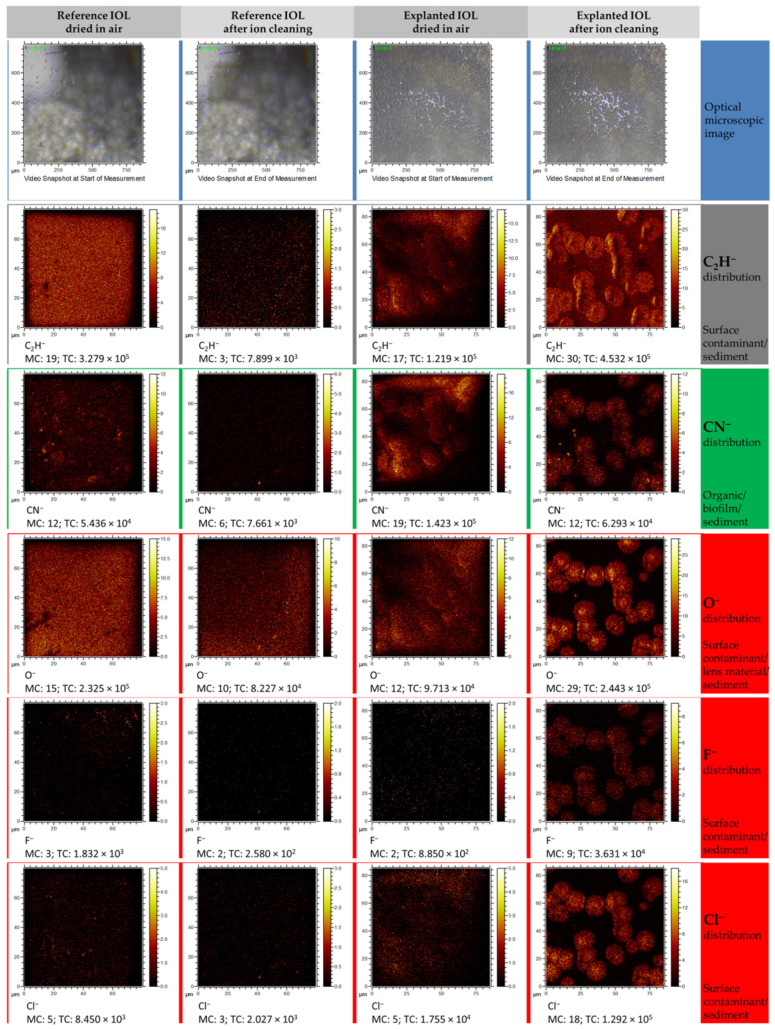
TOF–SIMS distribution maps of selected elemental and molecular ions obtained from measurements in negative polarity. Each column represents a set of distribution maps of CN^−^, O^−^, F^−^, and Cl^−^. The first and second columns represent the ions’ distribution for the reference lens before and after the cleaning procedure, respectively, whereas the third and fourth columns show before and after cleaning of the explanted lens, respectively.

In the case of chlorine or fluoride distribution maps, obtained from measurements performed in the negative polarity mode (see Figure 8), the image resembles the distribution of the Ca^+^ and Na^+^ ions. Small amounts (Cl^−^, O^−^) or no (F^−^) were observed on the contaminated surfaces of both IOLs, and after surface cleaning, an increase of those ions was observed in the regions where the sediment is present. The studies carried out in the negative polarity also allowed to determine that carbon-based compounds (represented here by the CN^−^ and C_2_H^−^ ion) are found in the observed round structures. This observation could suggest the presence of organic compounds in the studied structures and is in good agreement with results obtained from the XPS analysis.

### 3.3. Sedimentation Mechanism

The mechanism of sediment formation on hydrophilic IOLs is being extensively studied. The literature on the subject discusses many cases of sedimentation, and also includes some ideas to describe this phenomenon [1,3,4,5,6,7,8,9,24,26,27,28,29,36,37,38,39,40,41]. Scherer, N C. D et al. in recent investigations [61] by detailed statistical analysis of a series of 223 explanted Lentis LS-502-1 IOLs revealed as a calcification risk factor an age at implantation. The direct cause of calcification is associated with the interplay between IOLs’ material characteristics, contamination resulting from manufacturing, and patient individual conditions [61]. In turn, comorbidities and the presence of surgery subsequent to the lens implantations are suspected as causes of secondary calcification in lenses explanted from polypseudophakic eyes [62].

The exact mechanism of the sediment formation on IOLs’ surfaces is not well established. The variety of lens types, lens designs, and environmental factors (lens contamination or patient condition) complicates the determination of a consistent model of the sediments formation. Such a general mechanism, without statistical analysis of a large number of cases, is unlikely to be determinable. However, considering the low probability of such sediments’ appearance, it is relevant to report each case. Moreover, detailed physicochemical analysis of a single case, explanted C-Flex lens after DSAEK, provided in this work allowed us to draw some useful conclusions and propose a fairly simple model of sediment formation on the tested lens.

**Figure 9:**
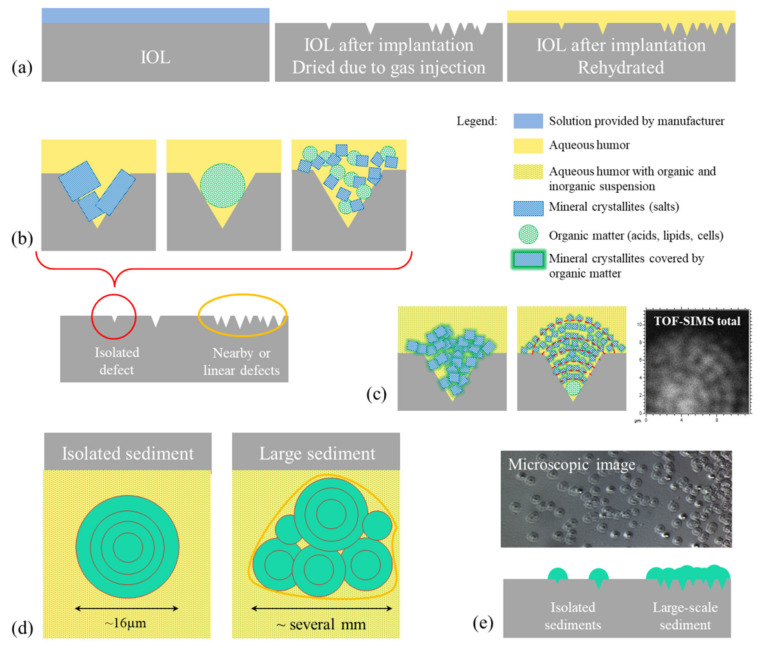
Diagrams illustrating the probable mechanism of sediment formation on IOL surface. (**a**) Scheme of lens cross sections at different stages presented in the following order: stored in the liquid foreseen by the manufacturer, after contact with the air bubble after DSAEK procedure, and after rehydration in eye. The probable origins of stages of sediment growth are shown (**b**) based on the literature and (**c**) proposed in this work. The sediment expansion was illustrated on scheme (**d**) top view and (**e**) cross-section view, combined with microscopic image and TOF–SIMS distribution maps of all detected ions.

On the basis of the results of our research and reports known in the literature, we present a diagram (see Figure 9) of the probable mechanism of opacification of the C-Flex lens caused by deposits formed on its surface. As results from the analysis of cases described so far, the damage to the lens is caused by drying of its surface after the DSAEK procedure, particularly when the air injection is repeated [5,6,7,8,9,11,12,13,14,22,23,24,26,27,28,29]. The literature reports also indicate that the source of sediment growth can be related to the altered blood–aqueous barrier, which could allow protein penetration into the IOL surface. For instance, the scanning electron microscopy (SEM) image obtained by Dhital et al. [6] seems to indicate that sediment grows into the IOL surface. However, because a large number of reported lens opacification cases were observed for patients after DSAEK, we believe that lens dehydration is a crucial step in the sedimentation process. In addition to literature reports, it is also supported by the fact that, in the case of the tested lens (C-flex 570C, Rayner Intraocular Lenses Ltd.), the manufacturer recommends that the lens should not be dried and should be implanted in the eye within 3 min of the time of folding or loading it into an injector [63]. It is thus understandable that prolonged contact with air can lead to changes of the lens surface. Here, a relatively long time, up to 2 days, of air bubble absorption took place; therefore, spot cavities or linear cracks are expected to appear on such IOLs’ surfaces. Topography studies of the surface of dried hydrophilic and hydrophobic lenses [64] showed that the surface roughness of the dried C-Flex lens is relatively high (around 14 nm) compared with other lenses, and that the C-Flex lens surface degeneration is rather related to point defects than linear defects. In Figure 9a, we present a schematic diagram of the suspected surface behavior under the conditions of storing the lens in its original box, during the DSAEK procedure and after the air bubble was absorbed and the lens was rehydrated in the eye environment. It is expected that the longer contact time of the lens surface with air will lead to increased degeneration of its surface due to the increased number of defects on its surface (see scheme Figure 9b of isolated defects, nearby point defects, and linear defects). In the place where the damaged surface of the lens comes into contact with the aqueous humor, nucleation processes of the sediment and its further growth occur. Firstly, the chemical composition of the lens material and polymer structure are most likely altered, thus influencing molecular interactions between the lens surface and aqueous humor. Secondly, the composition of the aqueous humor will surely vary from patient to patient and will depend on the patient’s state of health, possible infections, inflammations, or drugs taken. It seems quite likely that the material diluted in aqueous solution will settle in surface cavities (defects). This will be favored by both the shape of the defective surface and the molecular interactions between PMMA hydroxyl groups and aqueous humor components. Figure 9b presents general diagrams of sediment structures, including mineral and organic matter or mixtures thereof, such as those considered in many articles [29,65,66]. The analysis of the results obtained for the examined lens indicates the presence of mineral grains covered by or mixed with organic matter (Figure 9c), and this finding is supported by the following: (i) the shape of the sediments (see Figure 9c—TOF–SIMS total ion map and microscopic image on Figure 9e); (ii) distribution maps of Ca, Cl, Na, and CN ions; and (iii) detection by XPS of inorganic compounds CaCO_3_ and some compound with Cl-O bonds like in NaClO_3_.

The concentric shape and microstructure of the deposits (granularity) indicate their increase from a central point, which is probably a defect in the lens surface caused by its drying. Around this point, almost spherical grains about 1 µm in size accumulate (see microscopic image in Figure 4 and Figure 5 and TOF–SIMS total signal in Figure 9c). The most probable process of such growth is diffusion-limited aggregation. In the case of this study, as a result of random moves of organic and inorganic particles suspended in aqueous humor (for which diffusion is one of the main mechanisms that contribute to the formation of aqueous humor [67]), the particles aggregate and are attached to the closest cluster. The analysis of TOF–SIMS distribution maps (see Figure 7 and Figure 8, Figure S3 in Comment S4 and Figure S4a,b in Comment S5 of Supplementary Materials) indicate that inorganic matter plays a leading role in this process—at least from the point of view of sediment volume. It seems that organic matter acts as a sediment binder.

In this case, the organic phase would be gradually overgrown by the mixture of organic and inorganic phases (see Figure 9c—middle image with red dotted curves), which could explain the results observed in the SEM image [6]; however, the results presented in this work do not allow to resolve this issue.

The size of the sediment is most likely related to the residence time of the injected air in the eye. It is interesting to note that the average diameter of the isolated sediments is of about 16 µm (see Figure 4, Figure 5, and Figure 9d,e); smaller or bigger isolated sediments are not present. This would suggest that lens surface degeneration does not progress after rehydration in the eye environment. No additional nucleation sites were observed outside the maximum area covered by the air bubble, which seems to confirm our hypothesis of dehydration as the primary cause of surface defects leading to sedimentation. In the central area of the lens, which was exposed to drying for a longer time, a high density of grains is observed (see Figure 3 and Figure 4 and Figure 9b,d,e) and the deposits overlap, forming a continuous layer with a diameter of several millimeters.

**Figure 10:**
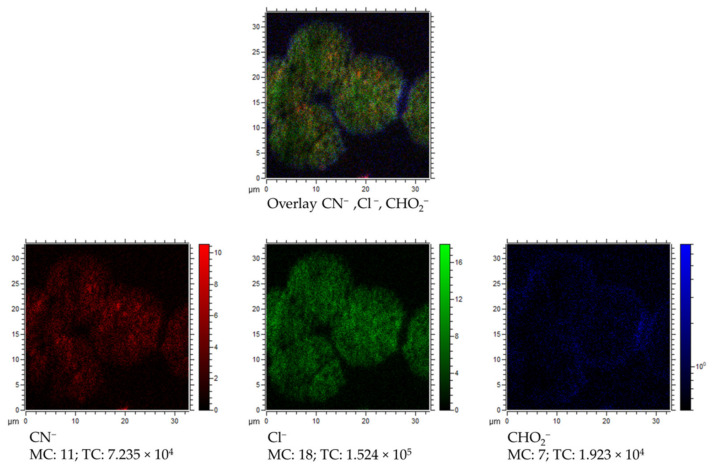
Overlay of TOF–SIMS distribution maps of CN^−^ (red), Cl^−^ (green), and CHO_2_^−^(blue) for partially covered with sediments surface of explanted IOL.

Additionally, we present the probable contribution of carboxylic group (most likely from PMMA or from some amino acids) in the IOLs’ sedimentation process. The XPS results indicate a presence of chemical bonds characteristic for that observed for PMMA (see Figure 6 O1s chemical state at about 533 eV) on the unused and explanted lens. In case of TOF–SIMS measurements, we observed that the COH_2_^−^ ion has a characteristic distribution, different from the ions associated with the sediments. In Figure 10, an overlay of the distribution maps of Cl^−^, CN^−^, and COH_2_^−^ together with separated distribution maps of each ion is presented. It is clearly visible that the COH_2_^−^ ion representing a carboxylic group is mostly located at the edges of the sediments. The intensity of this ion is relatively low inside and outside of the sediment area. It seems like some reaction or molecular interactions between the lens surface and sediment itself take place. Such interactions could be responsible for further damage of the lens, explaining the appearance [6] of IOLs’ pits. It is also worth mentioning that some organic compounds (related to the detected CN^−^ ions, see Figure 10 CN^−^) have a slightly different location in the sediment than Cl^−^ related to the mineral phase (see Figure 10 Cl^−^) of the sediment. This would suggest that the organic components of the aqueous humor participate in sediment formation in various manners.

Opacification of hydrophilic acrylic IOLs following intraocular gas injection has been described not only after intracameral injection of air or gas. Several reports described the opacification occurred after intravitreal gas injection during pars plana vitrectomy (PPV), the surgical procedure performed for treating diseases of the posterior segment of the eye [6,23,32,36,68,69,70,71,72]. The typical appearance of the opacification in most cases was similar to that occurring after DSAEK/DMEK [6,17,23,32,36,62,68,69,70,71,72]. Because the opacification appears mainly on the anterior surface of the IOL, although gas is injected into the vitreous, it is presumed that gas is likely to migrate into the anterior chamber through damaged zonular fibers [70]. Laboratory analysis demonstrated deposition of calcium and phosphate on IOL surface, as in the cases of IOL opacification after DSAEK [6,23,32,36,68,69,70,73].

There are many common morphological features for IOL opacification after inracameral and intravitreal gas injection. However, it is very likely that other factors, mainly an exacerbated inflammatory response due to repeated breakdown of the blood–aqueous barrier after multiple intraocular procedures, are more important in the development of this complication after the posterior segment procedures [6,23,35,36]. In our study, we focused on the analysis of IOL opacification after DSAEK, thus we have no evidence that our model is applicable to explain the post-PPV opacity mechanism. The calcification of a hydrophilic intraocular lens after pars plana vitrectomy due to the exposure of the lens surface to gas was observed, which, through increased hydrolyzation of the polyacrylate, triggers biomineralization [72]. The conclusions contained in this publication are in good agreement our observations and the proposed model of the mechanism of nucleation and sediment growth.

### 3.4. Potential Impact on Current Medical Practice

Although the incidence of opacification of hydrophilic acrylic IOLs after DSAEK reaches 5–9% [9,10], it is worth noting that IOL replacement is much less frequently performed in eyes after DSAEK than in eyes with a healthy cornea, where it is a treatment of choice, with good visual results [15]. In patients with less advanced opacity, the management is often limited to observation only. Ahad et al. described a relatively large series of post-DSAEK eyes in which the IOL opacification was found in 15 of 154 eyes, but none of the IOLs were explanted [10]. Morgan-Warren et al. described the replacement of only one in a series of six opacified IOLs after DSAEK, which was performed along with the simultaneous exchange of the failed graft [14]. Park et al. published a study on a similarly large group in which only one of the five IOLs with opacification after DSAEK was exchanged because of patient’s severe visual impairment [4]. Thus, the described principles of management determine the limited availability of samples for testing, which results in the main limitation of our work—the analysis was made for single explanted IOL and for the reference IOL.

The deleterious effect on visual acuity and quality of vision, a proven relationship with intraocular instillation of air or gas, and the lack of effective treatment fully justify the currently recommended treatment to reduce the incidence of the complication.

The most obvious, simplest, and generally accepted principle is avoiding hydrophilic acrylic IOL implantation during cataract surgery when it is combined with DSAEK or DMEK, and in patients with endothelial dystrophy who may probably require DSAEK or DMEK in the future.

However, this recommendation does not solve the problem of a certain group of pseudophakic patients qualified for DSAEK due to acquired endothelial dysfunction (pseudophakic bullous keratopathy, trauma, glaucoma drainage devices, or endothelial failure post penetrating keratoplasty) who already have hydrophilic acrylic IOL implanted. Some authors suggest shortening the length of the air tamponade to only 10 min [10]. This approach, nevertheless, does not seem to be a solution, because, although the authors did not observe this complication in their study, it can increase the risk of graft detachment and thus increase the frequency of re-bubbling. Other authors suggest to irrigate the anterior chamber with saline and leave it filled for a few minutes, in order to facilitate passive diffusion, and then remove the available calcium ions from the IOL. They believe that this would prevent the formation of calcium phosphate crystals during gas exposure [73]. So far, the effectiveness of such a surgical technique adjustment has been confirmed only in the in vitro model.

## 4. Conclusions

In this work, we report on the physicochemical studies of C-Flex lens explanted from the eye because of reduced visual acuity resulting from IOL opacification and corneal graft decompensation after endothelial keratoplasty. Detailed spectroscopic and AFM analyses of the reference and explanted IOL surfaces were carried out to detect a potential relationship between the physicochemical properties of the hydrophilic IOLs’ surface and the appearance of the sediment in connection with the DSAEK procedure.

Among the elements indicating significant changes in the chemical composition of the analyzed surfaces, nitrogen seems to be the most important. The analysis indicated the presence of heterocyclic aromatic organic compound (melanin and/or tryptophan) on the surface of the explanted IOL, which may appear as a result of the pathogenetic mechanism occurring in the eye [74,75]. Nevertheless, the presence of melanin on the surface of the IOL was most likely the result of mechanical damage to the iris epithelium as a result of iridectomy performed during DSAEK.

Looking at other results regarding the explanted IOL, one can also notice the slight increase of silicon, indicating the presence of compounds typically associated with the procedure for the production of flexible materials. The presence of calcium, chlorine, and sodium ions, whose main source is probably the aqueous humor of the eye, is clearly revealed via TOF–SIMS measurements. Those elements were also observed in a small amount using energy-dispersive X-ray spectroscopy in the recent work of Werner [36].

The reduction of IOL transparency due to sediment formation appears to be a multi-stage process in which air injected into the anterior chamber while DSAEK acts as a catalyst. Thus, repeated intracameral injection due to displacement of the donor transplant (re-bubbling) in the early postoperative period after DSAEK extends the air–IOL contact time, which increases the possibility of IOL surface damage. The localization and morphology of present sediment is very likely related to dehydration of the IOL surface due to its surface exposure to applied gas, which is oxygen here. Such dehydration would be a cause of microdegradation of the hydrophilic IOL surface, which could constitute a good substrate for sediment deposition. The oxygen contained in the air bubble applied during posterior lamellar keratoplasty most likely participates in processes occurring at the air–IOL interface. Regarding the dynamics of sediments’ growth, it is noteworthy that the grains are almost the same size (diameter is 16 µm based on AFM measurements) and there are no smaller grains. This means that the process of their growth began almost at the same time as the air bubble disappeared (it is absorbed after several hours after injection). In turn, the density of the grain distribution is the result of the amount of surface damage caused by contact with air in the bubble area. In the central part of the explanted lens, where an indiscrete sediment layer was observed, the contact with air was the longest, thus the surface was to a greater extent initially damaged. Further degeneration of the lens surface is abruptly stopped by rehydration of the lens surface, thus no new, smaller size deposits are observed on the explanted lens surface. To conclude, the results of the C-Flex lens analysis indicate a high similarity of observed sediments to secondary calcifications described in the literature [5]. The secondary type of calcification is dominant in the examined case; however, because of the presence of trace elements (Ca, Na) on the reference lens surface, primary calcification should also be considered.

The application of air bubble during the implantation and the resulting dehydration causes degeneration of the IOL surface, thus changing the physicochemical properties of the lens surface. Manufacturing of IOLs is also considered, but to a lesser extent, as a factor causing surface degeneration. Precipitation from aqueous humor solution and sedimentation of mineral and organic deposits seem to be specific for the patient eye environment. The given statements are also the conclusion of a recent review discussing the potential causes of opacity observed for various types of IOLs [41].

As has been shown, currently existing clinical recommendations do not allow for the complete elimination of the problem of IOL opacification, and it is expected that it will continue to be reported. That is why it is important to know the mechanisms leading to this serious complication as accurately as possible. The simultaneous application of several research techniques in the presented work allowed to create a hypothetical model of this complication, and may contribute to understanding the complex physical and biochemical mechanisms underlying this phenomenon.

## Supplementary Materials

The following are available online at https://www.mdpi.com/1996-1944/13/18/4145/s1, Comment S1: XPS and TOF–SIMS technical and analytical specifications in the context of chemical analysis and detection of particular compounds. Comment S2. Chemical composition comparison between reference and explanted IOL derived from XPS analysis. Comment S3. TOF–SIMS mass spectra obtained from explanted IOL before surface cleaning. Comment S4. TOF–SIMS distribution maps of selected elements (few additional ions were added) obtained from explanted lens after surface cleaning and the hydroxyapatite issue Comment S5. Growth mechanism discussed through the analysis of TOF–SIMS (a) overlay of O^−^, Cl^−^, and CN^−^ distribution maps and (b) distribution maps of all ions detected from explanted IOL and Ca^+^ and Na^+^. Figure S1. Cross section of explanted IOL. Figure S2. Mass spectra of explanted IOL. Figure S3. Distribution maps of selected ions obtained from TOF–SIMS measurements performed in negative polarity. Figure S4. (a) Overlay of CN^−^, Cl^−^, and O^−^ distribution maps for TOF–SIMS measurements in negative polarity. (b) Total ion distribution maps for 35 × 35 µm (cluster of sediments) and 12 × 12 µm (part of single sediment) regions as well as Na+ and Ca+ distribution maps from 12 × 12 µm region. Table S1. Technical specifications of applied techniques. Table S2. Chemical composition of examined IOLs. Table S3. List of characteristic for PMMA, methionine, tryptophan, valine ions.
