# Supplementary material for: Physicochemical Analysis of Sediments Formed on the Surface of Hydrophilic Intraocular Lens after Descemet’s Stripping Endothelial Keratoplasty
Source: Materials (Basel). 2020 Sep 17;13(18):4145. doi: 10.3390/ma13184145 (PMC7560278; doi:10.3390/ma13184145)
Supplement: Supplementary file 1 [file materials-13-04145-s001.pdf]

Supplementary materials

Physicochemical analysis of sediments formed on the surface of hydrophilic intraocular lens after Descemet's stripping endothelial keratoplasty

Dorota Tarnawska [1,2], Katarzyna Balin [3,*], Maria Jastrzębska [3], Agnieszka Talik [3], and Roman Wrzalik [3]

1 Institute of Biomedical Engineering, Faculty of Science and Technology, University of Silesia, 41-200 Sosnowiec, Poland; dorota.tarnawska@us.edu.pl
2 Department of Ophthalmology, District Railway Hospital, Panewnicka 65, 40-760 Katowice, Poland
3 August Chełkowski Institute of Physics, Faculty of Science and Technology, University of Silesia, 41-500 Chorzów, Poland; katarzyna.balin@us.edu.pl (K.B.); maria.jastrzebska@us.edu.pl (M.J.); agnieszka.talik@smcebi.edu.pl (A.T.); roman.wrzalik@us.edu.pl (R.W.)
* Correspondence: katarzyna.balin@us.edu.pl

Received: 1 July 2020; Accepted: 14 September 2020; Published: 17 September 2020

Comment 1. XPS and TOF-SIMS technical and analytical specifications in the context of chemical analysis and detection of particular compounds.

Table S1. Technical specifications of applied techniques.

	XPS	TOF-SIMS
Sampling volume	3-5 nm	below 1 nm
Detected elements	Li-U	H-U, molecular species
Detection limits	0.01–1 at%	10^7–10^{10} at/cm^2 (depend on element, and matrix/surrounding)
Atomic concentration	Yes	No
Chemical bonds	Yes	not directly, however molecular species are detected
Imaging	not in performed experiment	Yes

The procedure proposed in the paper for the examination of unused and explanted IOLs (as described in 2.3.4 section) is the optimal procedure combining the XPS and TOF-SIMS measurements on single lens. However, it limits the field of chemical analyzes offered by the XPS spectrometer. Here (to set the attention, let's focus on the explanted lens), we worked with one explanted lens that had to be measured on XPS, TOF-SIMS and AFM and in which deposits cover not uniformly ~3-4 mm2 area (additionally curved shape of the IOL creates additional measurement difficulties and limits available for analysis surface). In order to analyze chemical composition and morphology of the lens surface and sediment at the same time, for all techniques, we selected areas of the explanted IOL surface that are partially covered with sediment. In a manuscript area represented by simplified scheme (see Figure 2 and Figure S1a), here by more realistic one (Figure S1b).

Among applied techniques TOF-SIMS, due to possibility of simultaneous chemical composition analysis and imaging was selected as the technique for which measurements were performed before and after sample surface cleaning. Because XPS has a larger sampling volume than TOF-SIMS (see table and graph above), probing the sediment composition was considered as possible even through the layer of surface contaminations.

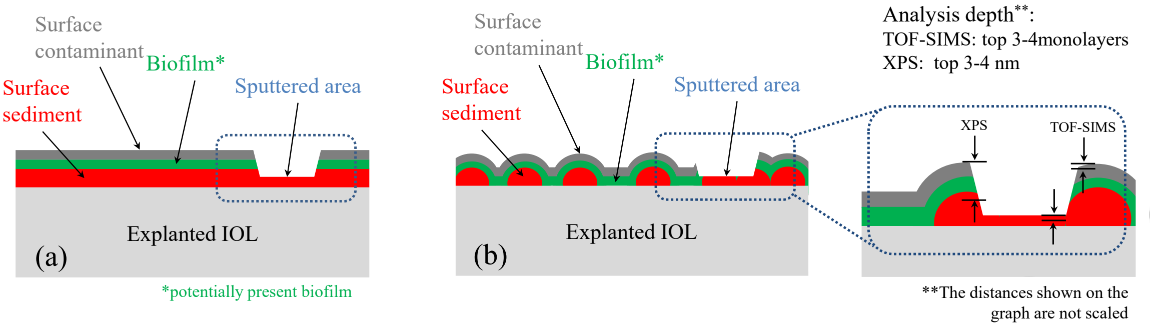

Figure S1. Cross section of explanted IOL, (a) simplified, (b) detailed scheme.

Performing additional XPS measurements (after cleaning of the lens surface) after the AFM and TOF-SIMS measurements was not considered, mostly due to possible surface damage by microscopic and spectroscopic examination. Other issues were considered important: contamination with Bi and Cs (from TOF-SIMS) as well as small probability of finding the same area of analysis for the XPS studies before and after surface cleaning. From the point of view of XPS we look at the sediments through the thin layer of surface contaminant and potentially present on lens surface biofilm. For this reason, the signal from the sediment will be weaker (see sampling volume in above table, and atomic concentration table in Comment 2). The fact that for example the expected hydroxyapatite was not detected could results from this lens specific case, or from presence of this compound in deeper part of the sediment. If focus only on the XPS measurements it would be recommended to perform the measurements before and after surface etching. This would bring more precise information about the composition of the sediment (elemental and chemical bonds).

Comment 2. Chemical composition comparison between reference and explanted IOL derived from XPS analysis.

Table S2. Chemical composition of examined IOLs.

	Reference IOL	Explanted IOL
Detected elements* (at. % - calculated atomic concentration)	C (69.53), O (29.04), N (0.64), Na (0.22), Si (0.08), F (0.48)	C (68.69), O (22.49), N (5.71), Na (0.46), Si (2.11), S (0.20), Cl (0.16), Ca (0.18)
Detected compounds or chemical bonds	• C-O, C=O, C-O=C bonds<br>• Poly (methyl methacrylate)/ Acrylic glass<br>• Amine<br>• Silicone oxide<br>• Silicone nitride<br>• Surface contaminants	• C-O, C=O bonds<br>• Poly (methyl methacrylate)/ Acrylic glass<br>• Amine<br>• Silicone nitride<br>• Silicone oxide<br>• Surface contaminants<br>• $CaCO_3$<br>• Compound with Cl-O bond as in $NaClO_3$<br>• C-N bond as in Polyaniline, or pyrrole ring<br>• SO_2<br>• Phenylene polysulfide

*Other elements have not been detected, however one has to remember that XPS is surface sensitive technique with specified detection limits (see table in Comment 1). The element identification and atomic concentration analysis were performed using Multipack software.

Comment 3. TOF-SIMS mass spectra obtained from explanted IOL before surface cleaning.

The extracted IOL was measured using TOF-SIMS spectrometer after the XPS measurements. As described in experimental part the surface of the explanted IOL was not cleaned during the XPS measurements. Thus presented below data are related to XPS investigation, as exactly the same surface is analyzed. The analysis was performed on part of the IOL that was not fully covered with the sediment, thus signal from the IOL can be observed. Positive mass spectra collected from explanted IOLs surface are presented in 0-120 Da mass range. Characteristic peaks for methionine, tryptophan and valine (red) have been collected in the table below, together with signal derived from IOL material (black) and selected peaks related with detected elements (blue).

Table S3. List of characteristic for PMMA, methionine, tryptophan, valine ions.

Assignment	Mass [u]				
	Elemental ions	Mass of characteristic ions of			
		PMMA	Methionine	Tryptophan	Valine
H^+	1.0073				
C^+	11.999				
CH_3^+		15.0229			
NH_3^+			17.0262	17.0262	17.0262
NH_4^+			18.0359	18.0359	18.0359
Na^+	22.9928				
Mg^+	23.9841				
$C_2H_2^+$		26.0139			
$C_2H_3^+$		27.0227			
Si^+		27.9751			
CH_2N^+			28.0191	28.0191	28.0191
CHO^+		29.002			
$C_2H_5^+$			29.0393		29.0393
CH_4N^+			30.0345	30.0345	30.0345
P^+	30.9799				
CH_3O^+		31.0179			
S^+	31.9755		31.9755		
H_3S^+			34.994		
K^+	38.9642				
$C_3H_3^+$		39.0221		39.0221	
Ca^+	39.9614				
$C_2H_4N^+$			42.0335	42.0335	42.0335
$C_2H_3O^+$	43.0199				
$C_2H_6N^+$			44.05	44.05	44.05
CH_3S^+			46.9932		
$C_4H_3^+$				51.0218	

Assignment	Mass [u]				
	Elemental ions	Mass of characteristic ions of			
		PMMA	Methionine	Tryptophan	Valine
$C_4H_5^+$		53.0384		53.0384	
$C_3H_4N^+$			54.0347		
$C_4H_7^+$		55.0565			
$C_3H_6N^+$			56.0502	56.0502	56.0502
$C_3H_5O^+$		57.0361			
$C_3H_8N^+$			58.0641	58.0641	58.0641
$C_2H_3O^+$		59.0213			
$C_2H_5S^+$			61.0096		
$C_4H_4O^+$		68.0256			
$C_4H_8N^+$			70.0652		70.0652
$C_4H_7O^+$		71.0522			
$C_4H_{10}N^+$					72.0826
$C_3H_7NO^+$				73.0531	73.0531
$C_2H_4NO_2^+$			74.0203		
$C_5H_5O^+$		81.035			
$C_5H_8N^+$			82.0663		
$C_5H_{12}N^+$			86.1007	86.1007	86.1007
$C_5H_{11}O^+$		87.0846			
$C_7H_5^+$		89.035			
$C_6H_7O^+$		95.0534			
$C_5H_8O_2^+$		101.0634			
$C_4H_{10}SN^+$			104.056		
$C_8H_{11}^+$		107.0854			
$C_6H_7O_2^+$		111.0467			
$C_8H_7N^+$				117.0602	
$C_5H_{12}NO^+$					118.0732

Characteristic peaks for methionine, tryptophan and valine have been colored in red and marked together with signal derived from IOL material (black) and selected peaks related with sediment (blue). TOF-SIMS positive mass spectra are limited to 0-120 (u) mass range. The mass resolution (m/Δm) was of about 5000 and allowed with high accuracy assign all peaks.

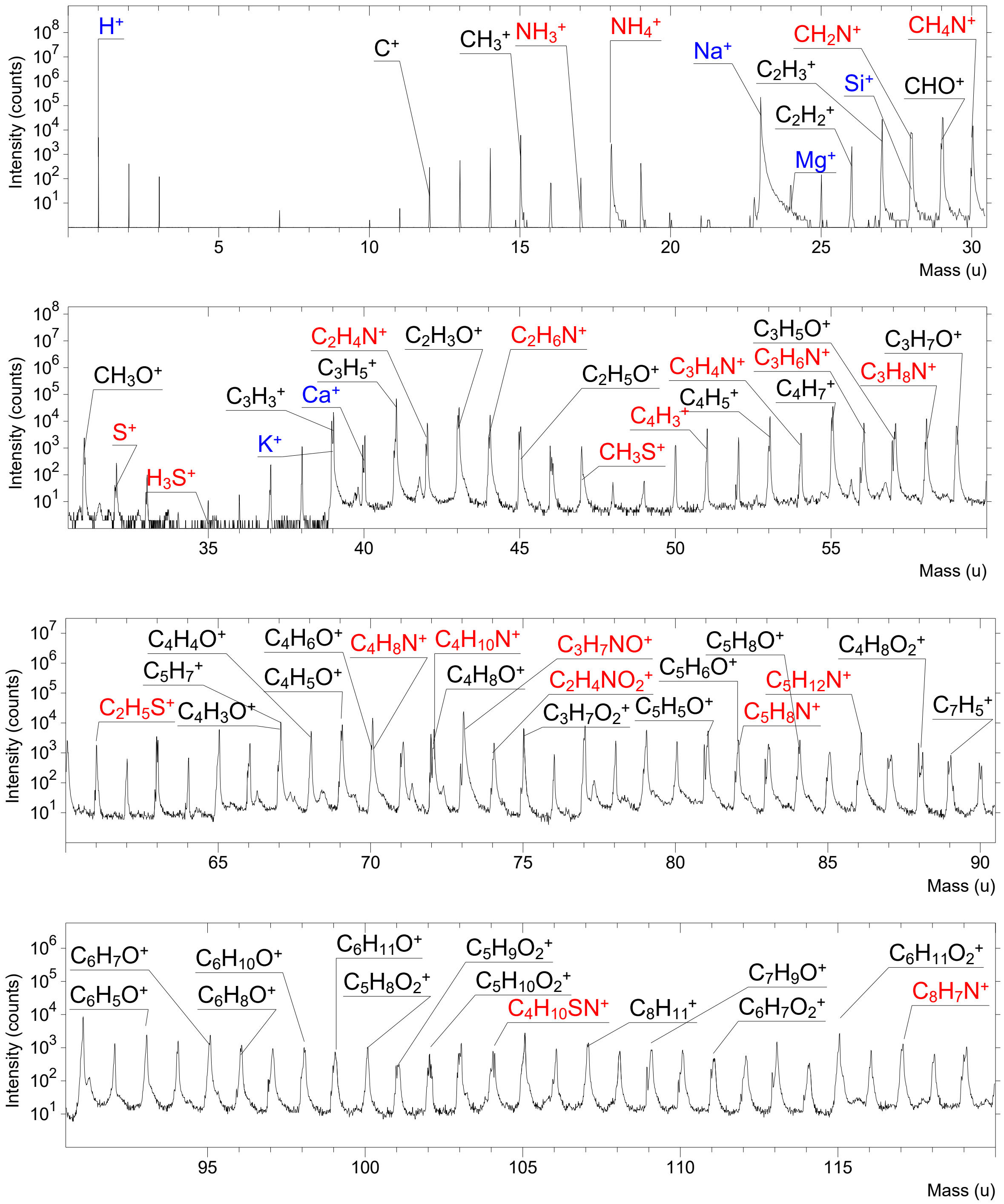

Figure S2. Mass spectra of explanted IOL.

Comment 4. TOF-SIMS distribution maps of selected elements (few additional ions have been added) obtained from explanted lens after surface cleaning and the hydroxyapatite issue.

Since the confirmation of hydroxyapatite was not possible by the meaning of the XPS measurements (P in preformed experiment was not detected, Ca exist as $CaCO_3$) we decided to look on the phosphorus related distribution maps obtained by TOF-SIMS after surface cleaning. Below, selected ion distribution maps (Figure S3), measured in negative polarity, have been presented for enlarged region which includes a cluster of several sediments (the region presented in the main body of the paper). The presence of P^-, PH^-, PO^- ions was confirmed, however, the signal intensities were surprisingly low, and therefore the intensity scale for P-related ions is presented on a logarithmic scale. Comparing with, for example, F^-, CN^-, O^-, Cl^- ions distribution maps the number

of detected P-related ions (TC—Total Counts) is lower by one or more orders of magnitude. The TOF-SIMS technique is not a quantitative technique—the number of counts cannot be directly related with the amount of particular element, however, seeing those results we are inclined to conclude that hydroxyapatite, even if present in the sediment, is not the main component of it.

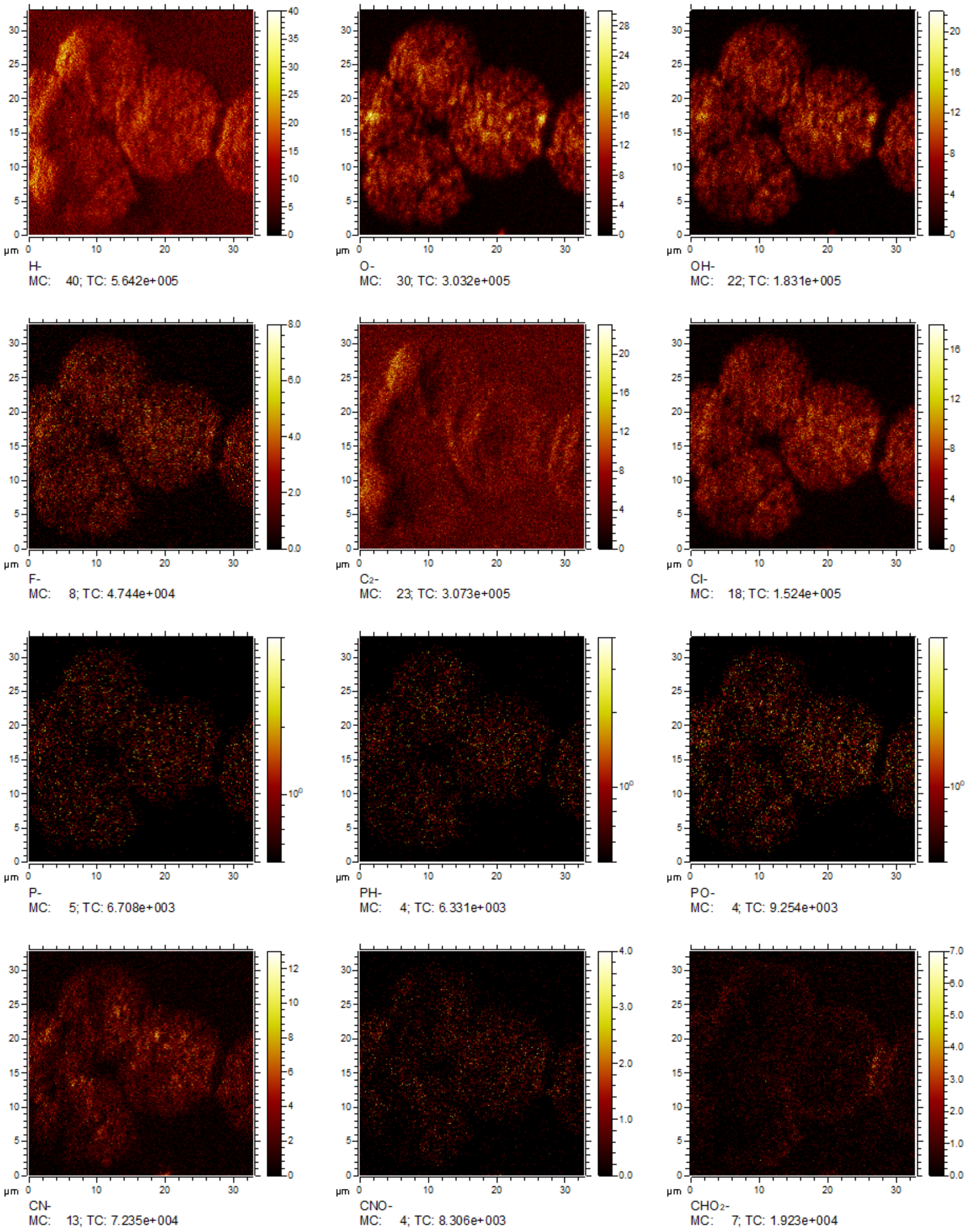

Figure S3. Distribution maps of selected ions obtained from TOF-SIMS measurements performed in negative polarity.

Comment 5. Growth mechanism discussed through the analysis of TOF-SIMS (a) overlay of O^-, Cl^-, CN^- distribution maps and (b) distribution maps of all ions detected from explanted IOL and Ca^+ and Na^+. For both cases the analysis is performed after surface cleaning.

In the main part of the article, we claim that inorganic matter is the main part of the sediment volume (see section 3.3). Analyzing maps of the distribution of individual ions, we can conclude that distribution of CN^- is different than Cl^- one; distribution of CN^- ions is rather unstructured, whereas on the distribution of Cl^- and O^- exhibit dendrite-like, circular structure (see Figure S4a).

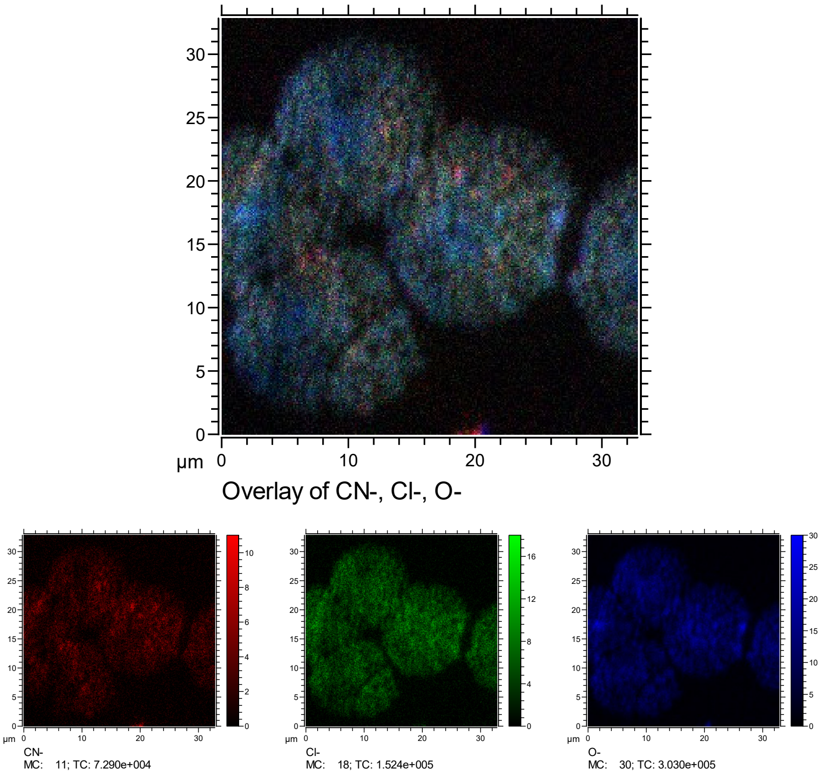

Figure S4a. Overlay of CN^-, Cl^-, O^- distribution maps for TOF-SIMS measurements in negative polarity.

The structured growth is even more pronounced if you taking a closer look at the maps of total ions and distribution of Na^+ and Ca^+, presented below (Figure S4b). Total secondary ion maps represent the signal obtained from all detected during the experiment secondary ions and image quite well mirrored the sediment morphology (compare with the AFM results presented on Fig. 5 of the main-body of the paper). When comparing those results with the distribution maps of Na^+ and Ca^+ (Figure S4b) it is obvious that those elements have an important role in the sediment growth. The results of the conducted research convince us that organic matter plays the role of sediment binder, while inorganic one occurs in the form of oxides and salts such as $CaCO_3$ and compound similar to $NaClO_3$ is the main part of the sludge (the presence of these compounds was confirmed in XPS measurements).

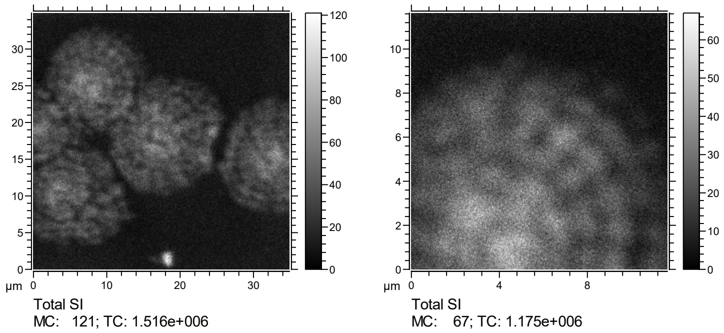

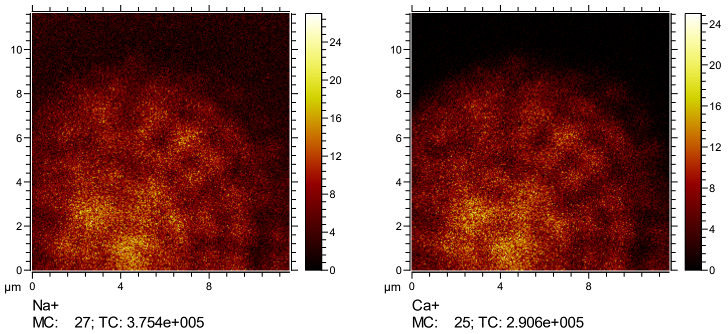

Figure S4b. Total ion distribution maps for 35 × 35 μm (cluster of sediments) and 12 × 12 μm (part of single sediment) regions as well as Na^+ and Ca^+ distribution maps from 12 × 12 μm region.
